# Aggressive mature natural killer cell neoplasms: from epidemiology to diagnosis

**DOI:** 10.1186/1750-1172-8-95

**Published:** 2013-07-01

**Authors:** Margarida Lima

**Affiliations:** 1 Department of Hematology, Laboratory of Cytometry, Hospital de Santo António (HSA), Centro Hospitalar do Porto (CHP), Rua D. Manuel II, s/n, 4099-001, Porto, Portugal; 2 Multidisciplinary Unit for Biomedical Investigation (UMIB/ICBAS/UP), Porto, Portugal

**Keywords:** NK-cell Neoplasms, NK/T-cell Lymphoma, Nasal-type, Aggressive NK-Cell Leukemia, CD56, Neoplasias de células NK, Linfomas de células NK, tipo nasal, Leucemia agressiva de células NK, CD56

## Abstract

Mature natural killer (NK) cell neoplasms are classified by the World Health Organization into NK/T cell lymphoma, nasal type (NKTCL), aggressive NK-cell leukemia (ANKCL) and chronic lymphoproliferative disorders of NK-cells, the latter being considered provisionally. NKTCL and ANKCL are rare diseases, with higher prevalence in Asia, Central and South America. Most NKTCL present extranodal, as a destructive tumor affecting the nose and upper aerodigestive tract (nasal NKTCL) or any organ or tissue (extranasal NKTCL) whereas ANKCL manifests as a systemic disease with multiorgan involvement and naturally evolutes to death in a few weeks. The histopathological hallmark of these aggressive NK-cell tumors is a polymorphic neoplastic infiltrate with angiocentricity, angiodestruction and tissue necrosis. The tumor cells have cytoplasmatic azurophilic granules and usually show a CD45^+bright^, CD2^+^, sCD3^-^, cytCD3epsilon^+^, CD56^+bright^, CD16^−/+^, cytotoxic granules molecules^+^ phenotype. T-cell receptor genes are in germ-line configuration. Epstein-Barr virus (EBV) -encoded membrane proteins and early region EBV RNA are usually detected on lymphoma cells, with a pattern suggestive of a latent viral infection type II. Complex chromosomal abnormalities are frequent and loss of chromosomes 6q, 11q, 13q, and 17p are recurrent aberrations. The rarity of the NK-cell tumors limits our ability to standardize the procedures for the diagnosis and clinical management and efforts should be made to encourage multi-institutional registries.

## Abstract (pt)

**Resumo:**

As neoplasias de células *natural killer* (NK) maduras foram classificadas pela Organização Mundial de Saúde em três entidades: o linfoma de células NK/T tipo nasal (NKTCL), a leucemia agressiva de células NK (ANKCL) e as doenças linfoproliferativas crónicas de células NK, estas últimas consideradas uma entidade provisória. Os NKTCL e a ANKCL são doenças raras, mais prevalentes na Ásia, na América Central e na América do Sul. A maioria dos NKTCL tem uma apresentação extra-ganglionar, na forma de tumor destrutivo que atinge o nariz e o trato aerodigestivo alto (forma nasal) ou qualquer órgão ou tecido (forma extranasal). A ANKCL manifesta-se como uma doença sistémica que evolui para a morte em poucas semanas. Do ponto de vista histopatológico, estas neoplasias caraterizam-se por um infiltrado polimórfico, com angiocentricidade, destruição vascular e necrose tecidular. As células tumorais têm grânulos azurófilos no citoplasma e o seu imunofenótipo (CD45^+forte^, CD2^+^, sCD3^-^, cytCD3epsilon^+^, CD56^+forte^, CD16^−/+^, proteínas dos grânulos citotóxicos^+^) é caraterístico. Os genes que codificam para o recetor das células T estão em configuração nativa. As células tumorais expressam geralmente proteínas da membrana e ARN do vírus Epstein Barr, com um padrão sugestivo de uma infecção vírica latente tipo II. As alterações cromossómicas são complexas, e algumas, como deleções nos braços longos dos cromossomas 6, 11 e 13 e do braço curto do cromossoma 17, ocorrem de forma recorrente. A raridade dos tumores de células NK limita a nossa capacidade para uniformizar os procedimentos de diagnóstico e a abordagem clínica, sendo necessário desenvolver esforços para promover os registos multicêntricos.

## Introduction

Lymphoproliferative disorders of natural killer (NK) cells are rare diseases which account for less than 5% of all lymphoid neoplasms and comprise different clinical entities [1-17].

The World Health Organization (WHO) classification of tumors of hematopoietic and lymphoid tissues, updated in 2008, has made advances in their classification. Accordingly, three disease conditions originating from mature NK-cells were proposed based on their distinct clinical and pathological features [18]. These include two aggressive mature NK-cell neoplasms – extranodal NK/T cell lymphoma, nasal type (NKTCL) [19], and aggressive NK-cell leukemia (ANKCL) [20] – and one provisional entity – chronic lymphoproliferative disorders of NK-cells (CLPD-NK) [21] (Table 1). The first two entities are indexed individually in the 10th revision of the International Classification of Diseases (ICD-10) [22] and in the 3rd edition of the ICD for Oncology (ICD-O-3) [23], as well as in Orphanet databases [24]. In addition, two diseases were proposed in the past as originating from NK-cell precursors, based mainly on the blastic appearance and the CD56^+^ immature immunophenotype of the neoplastic cells. The first, NK-cell lymphoblastic leukemia / lymphoma [25], in fact comprise an heterogeneous group of immature disorders originating from NK-, T- and/or myeloid cell precursors, and is now being considered in the group of the acute leukemia of ambiguous lineage; the other, blastic plasmacytoid dendritic cell neoplasm, previously designed blastic NK-cell lymphoma, arises from plasmacytoid dendritic cells and should no longer be considered a NK-cell malignancy [26].

**Table 1:** Natural killer cell neoplasms according to the World Health Organization classification of tumors of hematopoietic and lymphoid tissues

**WHO classification, 2008**	**Disease entities**	**Putative cells of origin**	**Orphanet numbers**	**Synonyms in the Orphanet data base**	**ICD codes**		**Prevalence categories (/1.000.000)**	**[References]**
					**ICD-10**	**ICD-O-3**		
NK-cell neoplasms	Extranodal NK/T-cell lymphoma (NKTCL), nasal type	Mature NK-cells	86879	NK/T-cell lymphoma; Nasal T/natural killer-cell lymphoma; Angiocentric T-cell lymphoma; Lethal midline granuloma	C86.0	9719/3	<1-9	[19,22-24]
	Aggressive NK-cell leukemia (ANKCL)	Mature NK-cells	86873	Aggressive NK-cell leukemia; Aggressive NK-cell lymphoma; NK-cell LGL leukemia; NK-cell large granular lymphocyte leukemia	C94.7	9948/3	<1-9	[20,22-24]
Provisional entities	Chronic lymphoproliferative disorders of NK-cells (CLPD-NK)	Mature NK-cells	Not available	NK-cell LGL leukemia; NK-cell large granular lymphocyte leukemia (considered together with ANKCL)	Not available (C94.7)*	Not available (9831/3)**	Unknown	[21-24]
	NK-cell lymphoblastic leukemia / lymphoma	NK-, T- and/or myeloid precursor cells	Not available	Not available	Not available	Not available	Unknown	[22-25]

Abbreviations: *ICD-10* International Statistical Classification of Diseases and Related Health Problems (formerly designated International Classification for Diseases), 10th revision, 2010, World Health Organization (available in: http://apps.who.int/classifications/icd10/browse/2010/en; accessed in 2 February 2013); *ICD-O-3* International Statistical Classification of Diseases and Related Health Problems, for Oncology (formerly designated International Classification of Diseases for Oncology, 3rd edition, 2000, World Health Organization (available in: http://www.who.int/classifications/icd/adaptations/oncology/en/index.html; accessed in 2 February 2013); *WHO* World Health Organization.

Blastic plasmacytoid dendritic cell neoplasm, also known as CD4^+^ CD56^+^ hematodermic neoplasm and previously designed blastic NK-cell lymphoma (Orpha:86870; ICD-10: C86.4, still referred as blastic NK-cell lymphoma; ICD-O: 9727/3) arises from plasmacytoid dendritic cells and should no longer be considered a NK-cell malignancy [26].

* Considering the synonymous list, CLPD-NK, which include chronic NK-cell LGL leukemia cases, are considered together with ANKCL; ** According to the proposal of the WHO classification, 2008, CLPD-NK should be considered together with T-LGLL in the ICD-O.

Nasal type NKTCL, originates in nasal and extranasal organs and tissues and account for the majority of cases, with only exceptional cases presenting primarily in the lymph nodes. ANKCL manifests as a systemic disease with multiorgan failure and rapidly evolutes to death. The diagnosis of these aggressive NK-cell neoplasms is often difficult and requires both clinical suspicion and a differentiated laboratorial approach based in morphological, immunophenotypic and molecular studies.

We review the epidemiology and the clinical and laboratorial criteria for the diagnosis of NKTCL and ANKCL, with emphasis on tissue histology and on the morphological, immunophenotypic and genetic features of the neoplastic cells.

## Review

### Epidemiology

Both NKTCL and ANKCL are relatively frequent in Asia, Central and South America, but extremely rare in Europe and North America [1-17].

Extranodal NK/T-cell lymphoma, nasal type, and ANKCL are relatively frequent in Central American (e.g. Mexico, Guatemala), South American (e.g. Argentina, Brazil, Peru, Chile) and Eastern (e.g. Hong Kong, Japan, Korea) countries, where they may account for up to 10% of the non Hodgkin’s lymphoma (NHL), whereas very uncommon in North America and Europe, where they represent less than 1% of the NHL^a^[1,2,27-29]. Moreover, in series from the United States in which the ethnic background was recorded, most patients with NKTCL were of Asian or Hispanic descent [30]. Few epidemiological data is available in Europe, where its prevalence has been estimated to be lower than 1–9 cases / 1.000.000 inhabitants.

Aggressive NK-cell neoplasms are almost always associated to Epstein Barr Virus (EBV) and similarly to that occurring in Hodgkin’s lymphoma and nasopharyngeal carcinoma, the neoplastic NK-cells usually have a type II latency pattern, expression of EBV nuclear antigens (EBNA) and latent membrane proteins (LMP) being limited to EBNA-1, LMP-1, and LMP-2 [31]. In Asia, increase in the risk of developing nasal NKTCL have been described among crop producers and individuals exposed to pesticides [32], also having an increased risk to develop NHL in general [33,34].

The International Peripheral T-cell Lymphoma Project (IPTCLP) group reported a four-fold higher relative frequency of NKTCL among lymphomas in Asian countries compared to Western countries, ANKCL being rarer than NKTCL (Table 2). From the 136 cases of NK-cell neoplasms analyzed by this group, collected in different centers from various countries in North America, Europe, and Asia, only 2 (1.5%) corresponded to ANKCL, as compared to 127 cases of NKTCL, the remaining 7 cases being unclassifiable according to the WHO schema [35]. Comparatively, based on the Japanese survey of NK-cell neoplasms diagnosed from 1994 to 1998 [36], the NK-cell Tumor Study Group reported on a Japanese series of 172 NK-cell tumors, which included 22 ANKCL (12.8%) [37]. Few European series of NKTCL were published to date [38,39] and, in Europe, reports on ANKCL are limited to sporadic cases [40-43].

**Table 2:** Frequencies of NK/T-cell Lymphoma, nasal type, and aggressive NK-cell leukemia in previous published series

**Series**	**Origin**	**NKTCL**	**ANKCL**	**NKTCL + ANKCL**	**[References]**
NK-cell Tumor Study Group *	Asia (Japan)	**150** (87.2%)	**22** (12.8%)	**172** (100%)	[37]
		Nasal: 123 (82.0%)			
		Extranasal: 27 (18.0%)			
International Peripheral T-Cell Lymphoma Project (IPTCLP) group**	North America, Europe, and Asia	**127** (98.5%)	**2** (1.5%)	**129** (100%)	[35]
		Nasal: 92 (72.4%)			
		Extranasal: 35 (27.6%)			
**Brazilian group**	South America (Brazil)	**120** (100%)	**0** (0%)	**120** (100%)	[44]
		Nasal: 97 (80.8%)			
		Extranasal: 23 (19.2%)			
**Intergruppo Italiano Linfomi**	Europe (Italy)	**26** (100%)	**0** (0%)	**26** (100%)	[39]
		Nasal: 23 (88.5%)			
		Extranasal: 3 (11.5%)			
**All series**		**423** (94.6%)	**25** (5.6%)	**447** (100%)	NA
		Nasal: 335 (79.4%)			
		Extranasal: 88 (20.6%)			

Abbreviations: *ANKTCL* aggressive NK-cell leukemia, *NKTCL* NK/T-cell Lymphoma, nasal type, *NA* not applicable.

* Most of the cases presented in this series were from the Japanese survey of NK-cell neoplasms diagnosed between 1994 and 1998, in which 237 cases were registered: 149 nasal-type NK-cell lymphoma (123 nasal and 26 extranasal), 22 aggressive NK-cell leukemia/lymphoma, 19 chronic NK lymphocytosis and 57 cases corresponding to diseases that are not considered as originating from mature NK-cells accordingly to the WHO classification updated in 2008 (11 myeloid/NK-cell precursor acute leukemia, 15 blastic NK-cell lymphoma, 21 precursor NK-cell acute lymphoblastic leukemia) [36].

** Consecutive cases of peripheral T-cell lymphoma (excluding Mycosis Fungoides and Sezary syndrome) and NK/T-cell lymphoma diagnosed between 1990 and 2002. Total number of cases registered: 1153 (Asia: 464, 40.2%; Europe and North America 689, 59.8%). Total number of NK-cell neoplasms registered: 136 (11.8%) (Asia: 104, 76.5%; Europe and North America: 32, 23.5%) (NKTCL: 127; ANKCL: 2; unclassified NK-cell neoplasms: 7).

Two variants of extranodal NKTCL, have been described, the nasal and the extranasal forms, the first being more frequent in nearly all reported series. In the register from the Japanese survey, only 18% of the NKTCL were extranasal [37], a higher percentage of extranasal cases (28%) being found among the NKTCL reported by the IPTCLP group [35]; in addition, a Brazilian and an Italian series of NKTCL included 19% and 12% of extranasal lymphomas, respectively [39,44] (Table 2).

### Clinical features

#### Extranodal NK/T cell lymphomas, nasal type

The nasal and extranasal forms of NKTCL differ from each other from the clinical point of view (Table 3) [1,10,14,45].

**Table 3:** Major clinical features of the NK-cell lymphoma, nasal type, and aggressive NK-cell leukemia

**Clinical features**	**NKTCL (nasal)**	**NKTCL (extranasal)**	**ANKCL**
Gender	Males > Females	Males > Females	Males = Females
Age (years)	50 – 60	50 – 60	30 – 40
Sites primarily involved	Nose, paranasal sinuses, orbits	Skin, gastrointestinal tract, salivary glands, lungs, eyes, soft tissues, adrenal glands, brain, breast, tong, other organs and tissues; rarely lymph nodes.	Blood, bone marrow, spleen, liver, lymph nodes
Clinical presentation	Nasal bleeding, nasal obstruction, palate perforation, mid-facial and/or upper airway destructive lesions	Ulcers, masses	Fever, jaundice, splenomegaly, hepatomegaly, lymphadenopathy, cytopenias, hemophagocytic syndrome
Prognosis	Early stages (I/II): good	Usually advanced stages (III/IV): poor	Highly aggressive / fatal
	Advanced stages (III/IV): poor		

Abbreviations: *ANKTCL* aggressive NK-cell leukemia, *NKTCL* NK/T-cell Lymphoma, nasal type.

Adapted from [14].

#### Nasal NK/T cell lymphomas

In contrast to that observed in Occidental countries, where the majority of the sinonasal lymphomas are B-cell lymphomas, in Asia more than 40% of these lymphomas originate from NK-cells. This neoplasm (also known as “lethal midline granuloma” or “midline malignant reticulosis”) commonly affects males and generally manifests as a localized disease, with mid-facial and/or upper airway destructive lesions [1,10,14,45]. Patients with nasal NKTCL present with nasal signals and symptoms, including mass, obstruction swelling, or bleeding. The tumor is locally invasive and often infiltrates the surrounding tissues, such as the nasopharynx, the oropharynx, the palate and the orbits; dissemination to other organs may occur in advanced disease stages.

#### Extranasal NK/T cell lymphomas

The extranasal form is frequently disseminated at the time of the diagnosis, most patients having multiple organs and tissues involved, usually without adenopathies [1,10,14,45]. Patients with extranasal NKTCL often have more adverse clinical features such as an advanced stage and poor performance status, and are more likely to have cytopenias, when compared to patients with nasal lymphoma [1,10,14]. The tumor may involve any anatomic site at the disease presentation or during disease progression, including the skin, the gastrointestinal tract, the testis, the lungs, the eyes, the soft tissues, the adrenal glands, the brain, the breast and the tongue [1,10,14]. The diagnosis of an extranasal NKTCL requires the exclusion of occult nasal disease, which may require nasal endoscopy with random biopsies.

Bone marrow involvement at the diagnosis is uncommon in NKTCL, in both nasal (<3.5%) and extranasal (<7%) cases [4,46]. In contrast, the hemophagocytic syndrome is relatively frequent, and often occurs in advanced disease [47].

#### Nodal NK-cell lymphomas, nasal type

Although nodal NKTCL are not being considered separately in the WHO classification, a few cases of NKTCL presenting primarily in the lymph nodes have been described [44,48-52]. Some of these cases were included in extranodal NKTCL series [44,50] and in series of patients with cytotoxic lymphomas [51]. For instance, in a review of 49 Asian cases of CD56+ neoplasms from which 34 were NKTCL, one had a primary nodal presentation [50] and a Brazilian series of 122 cases NKTCL, from which 23 cases were extranasal, included 6 nodal cases [44]. In another series of 66 patients with nodal cytotoxic cell lymphomas, one had the classic NKTCL phenotype [51]. In addition, cases of nodal lymphomas with a typical NKTCL phenotype and T-cell receptor (TCR) gamma (*TCRG*) gene rearrangements in germ-line configuration were described as case reports [48,49]. However, in other cases, the tumor probably originates from cytotoxic T-lymphocytes, as in the series of nodal lymphoma with a typical NKTCL phenotype reported by Takashi et al., from which 4 cases had clonal *TCRG* gene rearrangements [52]. Nodal NKTCL have a poor prognosis, most patients surviving for less than one year; they usually affect the cervical lymph nodes and the histology and phenotype are similar to those of extranodal NKTCL.

#### Aggressive NK-cell leukemia

Aggressive NK-cell leukemia is a very rare and extremely aggressive neoplasm, also with a higher prevalence among Asians [2,35,36,53-55]. Men and women are equally affected and the disease usually manifest in the third or four decades. Patients usually present extremely ill, with fever and other systemic symptoms, hepatosplenomegaly, pancytopenia and abnormal liver function. Serum levels of lactic dehydrogenase (LDH) and Fas Ligand (FasL) are often markedly increased. The hemophagocytic syndrome is frequent at diagnosis or during the disease course, resulting from uncontrolled monocyte/macrophage activation in response to cytokines produced by the neoplastic NK-cells [56-61]. The natural disease course is fulminant, with multiorgan failure and disseminated intravascular coagulation, death occurring usually within a few weeks [62].

### Clinical staging

The Ann-Arbor staging system, originally designed for Hodgkin’s lymphoma, is used for clinical staging of the NHL in general (Table 4) [63,64]. However, this system is not completely satisfactory for NKTCL, as it does not take into account the tumor size and the invasion to contiguous structures, which may be important prognostic features. Consequently, a modified tumor-staging system originally proposed for sinonasal B-cell lymphoma was adopted, which takes into account the local involvement [65] (Table 4).

**Table 4:** Clinical staging systems used for aggressive NK-cell neoplasms

**Staging system**	**Stages**	**Staging criteria**	**[References]**
Ann Arbor staging system*	Stage I	Confined to one lymph node site-	[63,64]
	Stage II	Confined to more than one lymph node site but on one side of the diaphragm.	
	Stage III	Confined to lymphatic tissue or spleen but on both sides of the diaphragm.	
	Stage IV	Bone marrow or liver involvement or extranodal sites with widespread involvement.	
Tumor staging used as a complement to the Ann Arbor staging system for nasal NKTCL	T1	Confinement to the nasal cavity.	[65]
	T2	Extension to the maxillary antra, anterior ethmoid sinus or hard palate.	
	T3	Extension to posterior ethmoid sinus, sphenoidal sinus, orbit, superior alveolar bone, cheeks, or superior buccinators space.	
	T4	Involvement of the inferior alveolar bone, inferior buccinators space, infratemporal fossa, nasopharynx, or cranial fossa.	

Abbreviations: *NK* natural-killer cells, *NKTCL* NK/T-cell Lymphoma, nasal type.

* Subscripts: A or B: absence (A) or presence (B) of constitutional symptoms; E: “extranodal” disease; X: largest tumor >10 cm large (“bulky disease”), or mediastinum wider than 1/3 of the chest on a chest X-ray; S: spleen involvement. NKTCL are usually extranodal lymphomas; thus, the subscript E applies to the vast majority of cases.

In order to perform disease staging, patients should be evaluated with routine hematological and biochemical analysis, bilateral bone marrow trephine biopsy, chest radiography, computerized tomography, and digestive endoscopy. In addition, magnetic resonance imaging helps to define the local involvement in nasal lymphoma, being superior to computerized tomography in determining the extent of soft-tissue infiltration, in differentiating inflamed from neoplastic tissue, and in clarifying bone lesions [66]. Positron emission tomography using fluorine-18-fluoro-deoxy-glucose is useful to investigate systemic spread and to distinguishing lymphoma from inflammatory masses [67].

The ratio of patients presenting limited extranodal disease stages (I_E_ or II_E_) versus those with presenting with advanced disease stages (III or IV) is 7:3 for nasal NKTCL and 4:6 for extranasal NKTCL [36].

### Laboratorial diagnosis

#### Histology and cytology

Natural killer/T cell lymphoma, nasal type, are histologically characterized by angiocentricity and invasion of the blood vessels by lymphoma cells, resulting in ischemic necrosis; the neoplastic cells have a variable size and usually appear morphologically immature and the cytological features are heterogeneous, with a variable mixture of inflammatory cells. Azurophilic granules are usually observed in the cytoplasm of the lymphoma cells using imprint smears [6]. Aggressive NK-cell leukemia cells are larger than normal large granular lymphocytes and often have a pale or slightly basophilic cytoplasm with azurophilic granules and a nucleus with slightly immature chromatin and inconspicuous or distinct nucleoli. As in NKTCL, necrosis, apoptosis, angioinvasion, and angiodestruction are common findings in tissue biopsies [6]. Also frequent is hemophagocytosis, which results from uncontrolled macrophage stimulation by inflammatory cytokines produced by the neoplastic cells and may result in the development of a hemophagocytic syndrome.

#### Immunophenotype

Most of our knowledge on the immunophenotype of the neoplastic NK-cells in NKTCL [30,35,37,50,68-70] as in ANKCL [2,50,53-55,71-75], is based on immunohistochemistry studies.

Like normal NK-cells, NKTCL and ANKCL tumor cells do not express CD3 and the TCR on their surface [1]. Nevertheless, they often have the epsilon chain of CD3 in the cytoplasm and, therefore, they may stain positively for CD3 in immunohistochemistry of paraffin sections, imposing the differential diagnosis with T-cell lymphoma [30]. Also as normal NK-cells, tumor NK-cells do not rearrange TCR genes, which can be shown to be in germ-line configuration by polymerase chain reaction (PCR) analysis. In addition, NKTCL and ANKCL tumor cells nearly always express CD2 and less often CD7 and CD8, but not CD4 and CD5. From the markers usually used to identify NK-cells, CD56 is the most frequently positive, whereas CD16 expression is variable, and CD57 is almost never found. In addition, cytotoxic granule–associated proteins such as T-cell–restricted intracellular antigen (TIA-1), granzyme B, and perforin are frequently expressed. The immunophenotypic characteristics of NKTCL and ANKCL cells seems to be similar, except for a higher frequency of cyCD3epsilon^+^ and a lower frequency of CD16^+^ cases in NKTCL [37].

Only a limited number of studies have evaluated the expression of killer receptors on the neoplastic NK-cells from patients with NKTCL and ANKCL [76-80]. Reverse transcriptase PCR techniques, revealed that most NKTCL tumor cells do express killer lectin type receptors (KLR) transcripts, including those for the CD94 and the NKG2 (most frequently NKG2A/B and NKG2D) receptors [80], a result that was confirmed by immunohistochemical stainings on tissue biopsies [76,77] and flow cytometry immunophenotyping of NKTCL and ANKCL cells [78]. The expression of killer immunoglobulin like receptors (KIR) seems to be more variable [76-78].

Linker for activation of T cells (LAT), a membrane protein that plays an important role in T-cell activation, which appears early during T-cell development and is present on T- and NK-cells, among others, is expressed in the great majority of T- and NK-cell neoplasms [81]. CD70, the receptor for CD27, was also found to be expressed both on NK-cell lines and on NKTCL lymphoma cells [82].

Ki67 is also frequently positive and the percentage of Ki67^+^ cells has proven to be a prognostic factor in extranodal NKTCL [83]. The Ki-67 protein (also known as MKI67) is a nuclear marker strictly associated with cell proliferation, which is present during all active phases of the cell cycle (G1, S, G2, and mitosis), but is absent from resting cells (G0) [84].

The fact that interactions between chemokines and chemokine receptors are involved in migration of lymphoma cells and tissue invasion have lead to the investigation of chemokine receptor expression on NKTCL cells [69,70,74,75]. These studies revealed that NKTCL cells usually express CXCR3, whose main ligand is CXCL11 (IP9, IFN-gamma inducible protein type 9) [68-70]. In addition, ANKL cells are simultaneously positive for CXCR1 and CCR5, whose major ligands are CXCL1 (interleukin-8, IL-18) and the CCL3 (MIP-1alpha, macrophage inflammatory protein type 1 alpha), CCL4 (MIP-1beta) and CCL5 (RANTES, regulated on activation, normal T cell expressed and secreted) chemokines, respectively [74,75].

Primary nodal NKTCL have the same phenotypic and genotypic characteristics as extranodal NKTCL, at least for the markers that are frequently tested, most of them being described as being CD2^+^, sCD3^-^, cytCD3epsilon^+^, CD56^+^, EBV^+^ and as having the TCR genes in germ-line configuration; moreover, they also usually have a CD4^-^, CD5^-^, CD7^-^, cytotoxic molecules^+^ phenotype [48,49,52].

In overall, the immunophenotypic features of the neoplastic NK-cells from patients with NKTCL and ANKCL are different from those of normal peripheral blood NK-cells [85], reactive NK-cells from patients with acute and chronic NK-cell lymphocytosis associated with viral infections and tumors [86,87], and monoclonal CLPD-NK [88].

#### Chromosomal and genomic abnormalities

Cytogenetic analyses of the NK-cell neoplasms are difficult because of the scarcity of specimens, small-size samples, tissue necrosis and the presence of inflammatory cells. Despite these difficulties, several studies were performed to date [89-96], some of them using comparative genomic hybridization and loss of heterozygosity techniques. Currently, genetic abnormalities specific for NKTCL and ANKL have not yet been identified, although complex chromosomal aberrancies occur in a large fraction of cases, abnormalities of the chromosome 6 being the most frequent finding [89]. In overall, cytogenetic aberrancies are seen in up to 77% of cases and karyotypic abnormalities observed include pseudodiploidy (57%), hyperdiploidy (30%), and hypodiploidy (13%) [89]. Recurrent abnormal chromosomal losses are 6q16–q25, 11q23.1, 11q24-q25, 13q14.11 and 17p13.3, among others; chromosomal gains include 1q21–q44, 2q13-q14, 2q31.1–q32.2, 6p25–p11.1, 7q11.2–q34, 7q35-q36 and 17q21.1 [89-96]. A common deletion on 6q in the target area 6q21-25 was identified, affecting multiple genes that are probably involved in oncogenesis and disease progression [90-96].

### Concluding remarks

Mature NK cell neoplasms comprise a wide spectrum of entities, from cases with an indolent disease course (chronic NK-cell lymphocytosis) to cases with aggressive clinical behavior (NKTCL and ANKCL). Aggressive NK-cell neoplasms are EBV-related diseases with a particular geographic distribution, have a typical immunophenotype and complex karyotypic abnormalities, often affecting extranodal organs and tissues, invading and destroying the adjacent structures, causing hemophagocytosis and disseminating thought the body. Due to their rarity, they are difficult to diagnose and to manage, and except for nasal NKTCL in early stages, they are refractory to the available therapies and have a very poor prognosis. Thus, multicentric registering studies and clinical trials are needed in order to better understand the disease biology and to develop new therapeutic agents.

## Endnotes

^a^ The estimated incidence of NHL varies worldwide, with the highest rates being reported in the most economically developed regions of the world (e.g. Northern America, Australia/New Zealand, and Northern Europe) and the lowest rates in the least developed regions (e.g. South-Central and Eastern Asia, and the Caribbean). According to data provided by the Cancer Research, UK (http://www.cancerresearchuk.org/, accessed in 10 September 2011), the crude incidence rate in the UK in 2009 was 22 new NHL cases for every 100,000 males and 18 for every 100,000 females and within the countries of the European Union, the highest age standardized incidence rates for 2008 were estimated to be in Luxembourg for men (around 19 cases per 100,000) and Ireland for women (more than 13 cases per 100,000), while the lowest rates were found in Greece for both sexes (each around 3 cases per 100,000).

## Abbreviations

ANKCL: Aggressive NK-cell leukemia; CCL3: C-C motif chemokine ligand type 3, also known as MIP-1alpha; CCL4: C-C motif chemokine ligand type 4, also known as MIP-1beta; CCL5: C-C motif chemokine ligand type 5, also known as RANTES; CCR5: C-C motif chemokine ligand type 5 (CD195); CLPD-NK: Chronic lymphoproliferative disorders of NK-cells; CXCL1: C-X-C motif chemokine ligand type 1, also known as IL-18; CXCL11: C-X-C motif chemokine ligand type 3, also known as IP9; CXCR1: C-X-C motif chemokine ligand receptor type 1 (CD181); CXCR3: C-X-C motif chemokine ligand receptor type 3 (CD183); EBNA: Epstein Barr virus nuclear antigens; EBV: Epstein-Barr virus; FasL: Fas ligand; ICD: International Classification of Diseases (now International Statistical Classification of Diseases and Related Health Problems); ICD-O: International Classification of Diseases for Oncology; IL-8: Interleukin-8 (CXCL1); IP9: IFN-gamma inducible protein type 9 (CXCL11); IPTCLP: International Peripheral T-cell Lymphoma Project; KIR: Killer immunoglobulin-like receptors; KLR: C-type lectin-like receptors; LAT: Linker for activation of T cells; LDH: Lactate dehydrogenase; LGL: Large Granular Lymphocytes; LMP: EBV-encoded latent membrane protein; MIP-1alpha: Macrophage inflammatory protein type 1 alpha (CCL3); MIP-1beta: Macrophage inflammatory protein type 1 beta (CCL4); NHL: Non-Hodgkin’s lymphoma; NK: Natural killer; NKTCL: NK/T cell lymphoma; PCR: Polymerase chain reaction; RANTES: Regulated on activation, normal T cell expressed and secreted (CCL5); TCR: T cell receptor; TIA-1: T-cell–restricted intracellular antigen; WHO: World Health Organization.

## Competing interests

The author discloses any financial and non-financial competing interests that may influence the interpretation of data or the presentation of information in the manuscript.

## Author’s contributions

ML reviewed the literature on the subject, had write and approved the final manuscript.

## Author’s information

ML is a senior medical doctor, responsible for the Laboratory of Cytometry of the Department of Hematology, Hospital de Santo António, Centro Hospitalar do Porto, Porto, Portugal. The Laboratory of Cytometry is a reference laboratory for the diagnosis of T- and NK-cell disorders. The author has been dedicated to the diagnosis and treatment of T- and NK-cell lymphoproliferative disorders and has already published a large number of papers in this field.

## References

[B1] Cheung MMC Chan JKC Wong K-F Natural killer cell neoplasms: a distinctive group of highly aggressive lymphomas/leukemias Semin Hematol 2003 40 221 232 12876671 10.1016/s0037-1963(03)00136-7

[B2] Suzuki R Suzumiya J Nakamura S Aoki S Notoya A Ozaki S Gondo H Hino N Mori H Sugimori H Kawa K Oshimi K Aggressive natural killer-cell leukemia revisited: large granular lymphocyte leukemia of cytotoxic NK cells Leukemia 2004 18 763 770 14961041 10.1038/sj.leu.2403262

[B3] Hasserjian RP Harris NL NK-cell lymphomas and leukemias: a spectrum of tumors with variable manifestations and immunophenotype Am J Clin Pathol 2007 127 860 868 17509983 10.1309/2F39NX1AL3L54WU8

[B4] Oshimi K Progress in understanding and managing natural killer-cell malignancies Br J Haematol 2007 139 532 544 17916099 10.1111/j.1365-2141.2007.06835.x

[B5] Aozasa K Takakuwa T Hongyo T Yang W-I Nasal NK/T-cell lymphoma: epidemiology and pathogenesis Int J Hematol 2008 87 110 117 18256789 10.1007/s12185-008-0021-7 PMC2276242

[B6] Liang X Graham DK Natural killer cell neoplasms Cancer 2008 112 1425 1436 18286525 10.1002/cncr.23316

[B7] Greer JP Mosse CA Natural killer-cell neoplasms Curr Hematol Malig Rep 2009 4 245 252 20425414 10.1007/s11899-009-0032-3

[B8] Harabuchi Y Takahara M Kishibe K Moriai S Nagato T Ishii H Nasal natural killer (NK)/T-cell lymphoma: clinical, histological, virological, and genetic features Int J Clin Oncol 2009 14 181 190 19593607 10.1007/s10147-009-0882-7

[B9] Kohrt H Advani R Extranodal natural killer/T-cell lymphoma: current concepts in biology and treatment Leuk Lymphoma 2009 50 1773 1784 19883307 10.3109/10428190903186502 PMC5568825

[B10] Gill H Liang RHS Tse E Extranodal natural-killer/t-cell lymphoma, nasal type Adv Hematol 2010 2010 627401 21234094 10.1155/2010/627401 PMC3018635

[B11] Suzuki R Treatment of advanced extranodal NK/T cell lymphoma, nasal-type and aggressive NK-cell leukemia Int J Hematol 2010 92 697 701 21116747 10.1007/s12185-010-0726-2

[B12] Aozasa K Zaki MAA Epidemiology and pathogenesis of nasal NK/T-cell lymphoma: a mini-review ScientificWorldJournal 2011 11 422 428 21336457 10.1100/tsw.2011.41 PMC5720017

[B13] Kobayashi S Natural killer cell leukemia: diagnosis 2011 InTech: Pathogenesis and Treatment. In Novel Aspects in Acute Lymphoblastic Leukemia. Edited by Faderl S

[B14] Yook-Lam K The diagnosis and management of extranodal NK/T-cell lymphoma, nasal-type and aggressive NK-cell leukemia J Clin Exp Hematop 2011 51 21 28 21628857 10.3960/jslrt.51.21

[B15] Tse E Kwong Y-L Treatment algorithms for mature T-cell and natural killer-cell neoplasms Future Oncol 2011 7 1101 1112 21919697 10.2217/fon.11.84

[B16] Jaccard A Hermine O Extranodal natural killer/T-cell lymphoma: advances in the management Curr Opin Oncol 2011 23 429 435 21743325 10.1097/CCO.0b013e328349aba6

[B17] Semenzato G Marino F Zambello R State of the art in natural killer cell malignancies Int J Lab Hematol 2012 34 117 128 21895989 10.1111/j.1751-553X.2011.01374.x

[B18] Swerdlow SH International Agency for Research on Cancer WHO classification of Tumours of Haematopoietic and lymphoid tissues 2008 Lyon: International Agency for Research on Cancer

[B19] Chan JKC Quintanilla-Martinez L Ferry JA Peh S-C Swerdlow SH, Campo E, Harris NL, Jaffe ES, Pileri SA, Stein H, Thiele J, Vardiman JW Extranodal NK/T-cell lymphoma, nasal type World Health Organization classification of Tumours of Haematopoietic and lymphoid tissues 2008 4 Lyon, France: International Agency for Research on Cancer (IARC) Press 285 288

[B20] Chan JKC Jane ES Ralfkiaer E Ko Y-H Swerdlow SH, Campo E, Harris NL, Jaffe ES, Pileri SA, Stein H, Thiele J, Vardiman JW Aggressive NK-cell leukaemia World Health Organization classification of Tumours of Haematopoietic and lymphoid tissues 2008 4 Lyon, France: International Agency for Research on Cancer (IARC) Press 276 277

[B21] Villamor N Morice WG Chan WC Foucar KK Swerdlow SH SH, Campo EE, Harris NL, Jaffe ES, Pileri SA, Stein H, Thiele J, Vardiman JW Chronic Iymphoproliferative disorders of NK cells World Health Organization Classification of tumours of haematopoietic and lymphoid tissues 2008 4 Lyon: International Agency for Research on Cancer (IARC) Press 274 275

[B22] World Health Organization The ICD-10 Classification of Mental and Behavioural Disorders: Clinical Descriptions and Diagnostic Guidelines 1992 Geneva: World Health Organization Available at: http://apps.who.int/classifications/icd10/browse/2010/en Accessed February 2, 2013

[B23] World Health Organization Fritz A, Jack A, Parkin DM, Percy C, Shanmugarathan S, Sobin L, Whelan L International classification of diseases for oncology, 3rd edition ((ICD-O-3) 2000 3 Geneva: World Health Organisation Available at: http://www.who.int/classifications/icd/adaptations/oncology/en/. Accessed February 2, 2013

[B24] Orphanet: an online database of rare diseases and orphan drugs Copyright, INSERM 1997. Available at: http://www.orpha.net/consor/cgi-bin/Disease_Classif.php. Accessed February 2, 2013

[B25] Borowitz MJ Bene ME Harris NL Porwit A Matures E Swerdlow SH, Campo E, Harris NL, Jaffe ES, Pileri SA, Stein H, Thiele J, Vardiman J Acute leukaemias of ambiguous lineage World Health Organization Classification of tumours of haematopoietic and lymphoid tissues 2008 4 Lyon, France: International Agency for Research on Cancer (IARC) Press 150 155

[B26] Pacchem F Jones DM Petrella T Swerdlow SH, Campo E, Harris NL, Jaffe ES, Pileri SA, Stein H, Thiele J, Vardiman JW Blastic plasmacytoid dendritic cell neoplasms World Health Organization Classification of Tumours of Haematopoietic and lymphoid tissues 2008 4 Lyon, France: International Agency for Research on Cancer (IARC) Press 145 147

[B27] Kwong Y-L Chan AC Liang R Chiang AK Chim CS Chan TK Todd D Ho FC CD56+ NK lymphomas: clinicopathological features and prognosis Br J Haematol 1997 97 821 829 9217183 10.1046/j.1365-2141.1997.1462962.x

[B28] Au W-Y Ma S-Y Chim C-S Choy C Loong F Lie AKW Lam CCK Leung AYH Tse E Yau C-C Liang R Kwong Y-L Clinicopathologic features and treatment outcome of mature T-cell and natural killer-cell lymphomas diagnosed according to the World Health Organization classification scheme: a single center experience of 10 years Ann Oncol 2005 16 206 214 15668271 10.1093/annonc/mdi037

[B29] Liu J Song B Fan T Huang C Xie C Li J Zhong W Li S Yu J Pathological and clinical characteristics of 1,248 non-Hodgkin’s lymphomas from a regional cancer hospital in Shandong, China Asian Pac J Cancer Prev 2011 12 3055 3061 22393989

[B30] Gaal K Sun NC Hernandez AM Arber DA Sinonasal NK/T-cell lymphomas in the United States Am J Surg Pathol 2000 24 1511 1517 11075852 10.1097/00000478-200011000-00006

[B31] Chiang AK Tao Q Srivastava G Ho FC Nasal NK- and T-cell lymphomas share the same type of Epstein-Barr virus latency as nasopharyngeal carcinoma and Hodgkin’s disease Int J Cancer 1996 68 285 290 8903467 10.1002/(SICI)1097-0215(19961104)68:3<285::AID-IJC3>3.0.CO;2-Y

[B32] Xu J-X Hoshida Y Yang W-I Inohara H Kubo T Kim G-E Yoon J-H Kojya S Bandoh N Harabuchi Y Tsutsumi K Koizuka I Jia X-S Kirihata M Tsukuma H Aozasa K Life-style and environmental factors in the development of nasal NK/T-cell lymphoma: a case–control study in East Asia Int J Cancer 2007 120 406 410 17066445 10.1002/ijc.22313

[B33] Miligi L Costantini AS Bolejack V Veraldi A Benvenuti A Nanni O Ramazzotti V Tumino R Stagnaro E Rodella S Fontana A Vindigni C Vineis P Non-Hodgkin’s lymphoma, leukemia, and exposures in agriculture: results from the Italian multicenter case–control study Am J Ind Med 2003 44 627 636 14635239 10.1002/ajim.10289

[B34] Chiu BC-H Weisenburger DD Zahm SH Cantor KP Gapstur SM Holmes F Burmeister LF Blair A Agricultural pesticide use, familial cancer, and risk of non-Hodgkin lymphoma Cancer Epidemiol Biomarkers Prev 2004 13 525 531 15066915

[B35] Au W-Y Weisenburger D Intragumtornchai T Nakamura S Kim W-S Sng I Vose J Armitage J Liang R Clinical differences between nasal and extranasal natural killer/T-cell lymphoma: a study of 136 cases from the International Peripheral T-Cell Lymphoma Project Blood 2009 113 3931 3937 19029440 10.1182/blood-2008-10-185256

[B36] Oshimi K Kawa K Nakamura S Suzuki R Suzumiya J Yamaguchi M Kameoka J Tagawa S Imamura N Ohshima K Kojya S Iwatsuki K Tokura Y Sato E Sugimori H NK-cell neoplasms in Japan Hematology 2005 10 237 245 16019472 10.1080/10245330400026162

[B37] Suzuki R Suzumiya J Yamaguchi M Nakamura S Kameoka J Kojima H Abe M Kinoshita T Yoshino T Iwatsuki K Kagami Y Tsuzuki T Kurokawa M Ito K Kawa K Oshimi K Prognostic factors for mature natural killer (NK) cell neoplasms: aggressive NK cell leukemia and extranodal NK cell lymphoma, nasal type Ann Oncol 2010 21 1032 1040 19850638 10.1093/annonc/mdp418

[B38] Kanavaros P Lescs MC Brière J Divine M Galateau F Joab I Bosq J Farcet JP Reyes F Gaulard P Nasal T-cell lymphoma: a clinicopathologic entity associated with peculiar phenotype and with Epstein-Barr virus Blood 1993 81 2688 2695 8387835

[B39] Pagano L Gallamini A Trapè G Fianchi L Mattei D Todeschini G Spadea A Cinieri S Iannitto E Martelli M Nosari A Bona ED Tosti ME Petti MC Falcucci P Montanaro M Pulsoni A Larocca LM Leone G NK/T-cell lymphomas “nasal type”: an Italian multicentric retrospective survey Ann Oncol 2006 17 794 800 16497823 10.1093/annonc/mdl015

[B40] Murdock J Jaffe ES Wilson WH McManus DT Alexander HD Morris TCMC Aggressive natural killer cell leukemia/lymphoma: case report, use of telesynergy and review of the literature Leuk Lymphoma 2004 45 1269 1273 15360011 10.1080/10428190310001646879

[B41] Guerrero AAM Lira VVP Bertin CP Galleguillos VM Ocqueteau TM Natural killer cell leukemia. Case report Rev Med Chil 2005 133 457 460 15953954 10.4067/s0034-98872005000400010

[B42] Castelli R Molteni M Gianelli U Cro L Grimoldi MG Cortelezzi A Aggressive natural killer cell leukaemia with a complex karyotype: a case report Ann Hematol 2006 85 66 68 16184393 10.1007/s00277-005-0001-4

[B43] Sousa J Cabezuelo L Almeida S Filipe C Simão A Carvalho A Nascimento Costa J Aggressive NK/T cell leukemia/lymphoma associated with EBV Acta Med Port 2011 24 Suppl 3 649 652 22856405

[B44] Gualco G Domeny-Duarte P Chioato L Barber G Natkunam Y Bacchi CE Clinicopathologic and molecular features of 122 Brazilian cases of nodal and extranodal NK/T-cell lymphoma, nasal type, with EBV subtyping analysis Am J Surg Pathol 2011 35 1195 1203 21716086 10.1097/PAS.0b013e31821ec4b5

[B45] Suzuki R Suzumiya J Oshimi K Differences between nasal and extranasal NK/T-cell lymphoma Blood 2009 113 6260 6261 author reply 6261–6262 19520819 10.1182/blood-2009-03-211011

[B46] Wong KF Chan JK Cheung MM So JC Bone marrow involvement by nasal NK cell lymphoma at diagnosis is uncommon Am J Clin Pathol 2001 115 266 270 11211616 10.1309/E5PR-6A9R-Q02N-8QVW

[B47] Takahashi N Miura I Chubachi A Miura AB Nakamura S A clinicopathological study of 20 patients with T/natural killer (NK)-cell lymphoma-associated hemophagocytic syndrome with special reference to nasal and nasal-type NK/T-cell lymphoma Int J Hematol 2001 74 303 308 11721967 10.1007/BF02982065

[B48] Chim C-S Ma ESK Loong F Kwong Y-L Diagnostic cues for natural killer cell lymphoma: primary nodal presentation and the role of in situ hybridisation for Epstein-Barr virus encoded early small RNA in detecting occult bone marrow involvement J Clin Pathol 2005 58 443 445 15790718 10.1136/jcp.2004.022608 PMC1770628

[B49] Chang S-T Liao Y-L Lin S-H Chuang S-S NK-cell lymphoma with nodal presentation and expression of cutaneous lymphocyte-associated antigen Pathol Res Pract 2010 206 463 466 19628338 10.1016/j.prp.2009.07.001

[B50] Chan JK Sin VC Wong KF Ng CS Tsang WY Chan CH Cheung MM Lau WH Nonnasal lymphoma expressing the natural killer cell marker CD56: a clinicopathologic study of 49 cases of an uncommon aggressive neoplasm Blood 1997 89 4501 4513 9192774

[B51] Kagami Y Suzuki R Taji H Yatabe Y Takeuchi T Maeda S Kondo E Kojima M Motoori T Mizoguchi Y Okamoto M Ohnishi K Yamabe H Seto M Ogura M Koshikawa T Takahashi T Kurita S Morishima Y Suchi T Nakamura S Nodal cytotoxic lymphoma spectrum: a clinicopathologic study of 66 patients Am J Surg Pathol 1999 23 1184 1200 10524519 10.1097/00000478-199910000-00003

[B52] Takahashi E Asano N Li C Tanaka T Shimada K Shimada S Yoshino T Kojima M Hara K Eimoto T Nakamura S Nodal T/NK-cell lymphoma of nasal type: a clinicopathological study of six cases Histopathology 2008 52 585 596 18370955 10.1111/j.1365-2559.2008.02997.x

[B53] Ruskova A Abizanda R Chan G Aggressive Natural Killer-Cell Leukemia: report of five cases and review of the literature Leuk Lymphoma 2004 45 2427 2438 15621755 10.1080/10428190400004513

[B54] Liu EB Chen HS Zhang PH Li ZQ Sun Q Yang QY Fang LH Sun FJ Aggressive NK-cell leukemia: report of nine cases and review of literature Zhonghua Xue Ye Xue Za Zhi 2006 27 116 119 16732967

[B55] Ryder J Wang X Bao L Gross SA Hua F Irons RD Aggressive natural killer cell leukemia: report of a Chinese series and review of the literature Int J Hematol 2007 85 18 25 17261497 10.1532/IJH97.A10612

[B56] Okuda T Sakamoto S Deguchi T Misawa S Kashima K Yoshihara T Ikushima S Hibi S Imashuku S Hemophagocytic syndrome associated with aggressive natural killer cell leukemia Am J Hematol 1991 38 321 323 1746541 10.1002/ajh.2830380412

[B57] Akashi K Mizuno S Epstein-Barr virus-infected natural killer cell leukemia Leuk Lymphoma 2000 40 57 66 11426629 10.3109/10428190009054881

[B58] Kaizu K Maeda M Ohkawa T Hayashida M Nakajima S Sugisaki Y Fukunaga Y Marked elevation of soluble fas ligand and cytokine secretion after splenectomy in aggressive natural killer cell leukemia/lymphoma Leuk Lymphoma 2004 45 2291 2294 15512819 10.1080/10428190412331283233

[B59] Choi Y-L Park J-H Kim W-S Lee D-Y Lee J-H Yang J-M Lee E-S Aggressive NK-cell leukaemia associated with reactive haemophagocytic syndrome Clin Exp Dermatol 2006 31 83 85 16309492 10.1111/j.1365-2230.2005.01986.x

[B60] Petterson TE Bosco AA Cohn RJ Aggressive natural killer cell leukemia presenting with hemophagocytic lymphohistiocytosis Pediatr Blood Cancer 2008 50 654 657 17853464 10.1002/pbc.21358

[B61] Suzuki S Uozumi K Utsunomiya A Ishitsuka K Masamoto I Owatari S Makino T White Y Arima N Aggressive NK cell leukaemia after splenectomy: association with CD95-resistant memory T-cell proliferation and recalcitrant clinical course of haemophagocytic syndrome Eur J Haematol 2008 81 236 241 18510705 10.1111/j.1600-0609.2008.01097.x

[B62] Han A-R Lee HR Park B-B Hwang IG Park S Lee SC Kim K Lim HY Ko YH Kim SH Kim WS Lymphoma-associated hemophagocytic syndrome: clinical features and treatment outcome Ann Hematol 2007 86 493 498 17347847 10.1007/s00277-007-0278-6

[B63] Carbone PP Kaplan HS Musshoff K Smithers DW Tubiana M Report of the committee on Hodgkin’s disease staging classification Cancer Res 1971 31 1860 1861 5121694

[B64] Lister TA Crowther D Sutcliffe SB Glatstein E Canellos GP Young RC Rosenberg SA Coltman CA Tubiana M Report of a committee convened to discuss the evaluation and staging of patients with Hodgkin’s disease: Cotswolds meeting J Clin Oncol 1989 7 1630 1636 2809679 10.1200/JCO.1989.7.11.1630

[B65] Robbins KT Fuller LM Vlasak M Osborne B Jing BS Velasquez WS Sullivan JA Primary lymphomas of the nasal cavity and paranasal sinuses Cancer 1985 56 814 819 4016673 10.1002/1097-0142(19850815)56:4<814::aid-cncr2820560419>3.0.co;2-p

[B66] Ooi GC Chim CS Liang R Tsang KW Kwong YL Nasal T-cell/natural killer cell lymphoma: CT and MR imaging features of a new clinicopathologic entity AJR Am J Roentgenol 2000 174 1141 1145 10749267 10.2214/ajr.174.4.1741141

[B67] Khong P-L Pang CBY Liang R Kwong Y-L Au W-Y Fluorine-18 fluorodeoxyglucose positron emission tomography in mature T-cell and natural killer cell malignancies Ann Hematol 2008 87 613 621 18509641 10.1007/s00277-008-0494-8

[B68] Schwartz EJ Molina-Kirsch H Zhao S Marinelli RJ Warnke RA Natkunam Y Immunohistochemical characterization of nasal-type extranodal NK/T-cell lymphoma using a tissue microarray: an analysis of 84 cases Am J Clin Pathol 2008 130 343 351 18701406 10.1309/V561QTM6854W4WAV

[B69] Ishida T Inagaki H Utsunomiya A Takatsuka Y Komatsu H Iida S Takeuchi G Eimoto T Nakamura S Ueda R CXC chemokine receptor 3 and CC chemokine receptor 4 expression in T-cell and NK-cell lymphomas with special reference to clinicopathological significance for peripheral T-cell lymphoma, unspecified Clin Cancer Res 2004 10 5494 5500 15328188 10.1158/1078-0432.CCR-04-0371

[B70] Yagi H Seo N Ohshima A Itoh T Itoh N Horibe T Yoshinari Y Takigawa M Hashizume H Chemokine receptor expression in cutaneous T cell and NK/T-cell lymphomas: immunohistochemical staining and in vitro chemotactic assay Am J Surg Pathol 2006 30 1111 1119 16931956 10.1097/01.pas.0000213267.92349.59

[B71] Falcão RP Rizzatti EG Saggioro FP Garcia AB Marinato AF Rego EM Flow cytometry characterization of leukemic phase of nasal NK/T-cell lymphoma in tumor biopsies and peripheral blood Haematologica 2007 92 e24 e25 17405750 10.3324/haematol.10654

[B72] Yoo E-H Kim H-J Lee S-T Kim W-S Kim S-H Frequent CD7 antigen loss in aggressive natural killer-cell leukemia: a useful diagnostic marker Korean J Lab Med 2009 29 491 496 20046078 10.3343/kjlm.2009.29.6.491

[B73] Liu E-B Chen H-S Zhang P-H Li Z-G Sun Q Yang Q-Y Fang L-H Sun F-J Clinicopathologic features of aggressive natural killer cell leukemia Zhonghua Bing Li Xue Za Zhi 2011 40 810 814 22336205

[B74] Makishima H Ito T Asano N Nakazawa H Shimodaira S Kamijo Y Nakazawa Y Suzuki T Kobayashi H Kiyosawa K Ishida F Significance of chemokine receptor expression in aggressive NK cell leukemia Leukemia 2005 19 1169 1174 15902300 10.1038/sj.leu.2403732

[B75] Makishima H Ito T Momose K Nakazawa H Shimodaira S Kamijo Y Nakazawa Y Ichikawa N Ueno M Kobayashi H Kitano K Saito H Kiyosawa K Ishida F Chemokine system and tissue infiltration in aggressive NK-cell leukemia Leuk Res 2007 31 1237 1245 17123604 10.1016/j.leukres.2006.10.020

[B76] Haedicke W Ho FC Chott A Moretta L Rüdiger T Ott G Müller-Hermelink HK Expression of CD94/NKG2A and killer immunoglobulin-like receptors in NK cells and a subset of extranodal cytotoxic T-cell lymphomas Blood 2000 95 3628 3630 10828054

[B77] Dukers DF Vermeer MH Jaspars LH Sander CA Flaig MJ Vos W Willemze R Meijer CJ Expression of killer cell inhibitory receptors is restricted to true NK cell lymphomas and a subset of intestinal enteropathy-type T cell lymphomas with a cytotoxic phenotype J Clin Pathol 2001 54 224 228 11253136 10.1136/jcp.54.3.224 PMC1731389

[B78] Mori KL Egashira M Oshimi K Differentiation stage of natural killer cell-lineage lymphoproliferative disorders based on phenotypic analysis Br J Haematol 2001 115 225 228 11722437 10.1046/j.1365-2141.2001.03038.x

[B79] Sawada A Sato E Koyama M Higuchi B Kusuki S Kim JY Takeshita Y Sakata A Sakata N Okamura T Yasui M Inoue M Kawa K NK-cell repertoire is feasible for diagnosing Epstein-Barr virus-infected NK-cell lymphoproliferative disease and evaluating the treatment effect Am J Hematol 2006 81 576 581 16823820 10.1002/ajh.20659

[B80] Nong L Zhang S Li Y Zhang Y Wang Y Li T Study on expression of natural killer (NK) cell C-type lectin-like receptors in nasal NK/T-cell lymphomas Zhonghua Bing Li Xue Za Zhi 2010 39 319 324 20654155

[B81] Facchetti F Chan JK Zhang W Tironi A Chilosi M Parolini S Notarangelo LD Samelson LE Linker for activation of T cells (LAT), a novel immunohistochemical marker for T cells, NK cells, mast cells, and megakaryocytes: evaluation in normal and pathological conditions Am J Pathol 1999 154 1037 1046 10233842 10.1016/S0002-9440(10)65356-4 PMC1866564

[B82] Yoshino K Kishibe K Nagato T Ueda S Komabayashi Y Takahara M Harabuchi Y Expression of CD70 in nasal natural killer/T cell lymphoma cell lines and patients; its role for cell proliferation through binding to soluble CD27 Br J Haematol 2013 160 331 342 23206232 10.1111/bjh.12136

[B83] Kim SJ Kim BS Choi CW Choi J Kim I Lee Y-H Kim JS Ki-67 expression is predictive of prognosis in patients with stage I/II extranodal NK/T-cell lymphoma, nasal type Ann Oncol 2007 18 1382 1387 17693651 10.1093/annonc/mdm183

[B84] Scholzen T Gerdes J The Ki-67 protein: from the known and the unknown J Cell Physiol 2000 182 311 322 10653597 10.1002/(SICI)1097-4652(200003)182:3<311::AID-JCP1>3.0.CO;2-9

[B85] Lima M Teixeira MA Queirós ML Leite M Santos AH Justiça B Orfão A Immunophenotypic characterization of normal blood CD56 + lo versus CD56 + hi NK-cell subsets and its impact on the understanding of their tissue distribution and functional properties Blood Cells Mol Dis 2001 27 731 743 11778657 10.1006/bcmd.2001.0443

[B86] Lima M Almeida J dos Anjos Teixeira M Queirós ML Justiça B Orfão A The “ex vivo” patterns of CD2/CD7, CD57/CD11c, CD38/CD11b, CD45RA/CD45RO, and CD11a/HLA-DR expression identify acute/early and chronic/late NK-cell activation states Blood Cells Mol Dis 2002 28 181 190 12064914 10.1006/bcmd.2002.0506

[B87] Lima M Almeida J Teixeira MA Santos AH Queirós ML Fonseca S Moura J Gonçalves M Orfão A Pinto Ribeiro AC Reactive phenotypes after acute and chronic NK-cell activation J Biol Regul Homeost Agents 2004 18 331 334 15786700

[B88] Lima M Almeida J Montero AG Teixeira M d A Queirós ML Santos AH Balanzategui A Estevinho A Algueró M d C Barcena P Fonseca S Amorim ML Cabeda JM Pinho L Gonzalez M San Miguel J Justiça B Orfão A Clinicobiological, immunophenotypic, and molecular characteristics of monoclonal CD56−/+dim chronic natural killer cell large granular lymphocytosis Am J Pathol 2004 165 1117 1127 15466379 10.1016/s0002-9440(10)63373-1 PMC1618630

[B89] Wong KF Zhang YM Chan JK Cytogenetic abnormalities in natural killer cell lymphoma/leukaemia–is there a consistent pattern? Leuk Lymphoma 1999 34 241 250 10439361 10.3109/10428199909050949

[B90] Siu LL Wong KF Chan JK Kwong YL Comparative genomic hybridization analysis of natural killer cell lymphoma/leukemia. Recognition of consistent patterns of genetic alterations Am J Pathol 1999 155 1419 1425 10550295 10.1016/S0002-9440(10)65454-5 PMC1866965

[B91] Ko YH Ree HJ Kim WS Choi WH Moon WS Kim SW Clinicopathologic and genotypic study of extranodal nasal-type natural killer/T-cell lymphoma and natural killer precursor lymphoma among Koreans Cancer 2000 89 2106 2116 11066052 10.1002/1097-0142(20001115)89:10<2106::aid-cncr11>3.0.co;2-g

[B92] Siu LL Chan V Chan JK Wong KF Liang R Kwong YL Consistent patterns of allelic loss in natural killer cell lymphoma Am J Pathol 2000 157 1803 1809 11106552 10.1016/S0002-9440(10)64818-3 PMC1885756

[B93] Zhang Y Matthiesen P Harder S Siebert R Castoldi G Calasanz MJ Wong KF Rosenwald A Ott G Atkin NB Schlegelberger B A 3-cM commonly deleted region in 6q21 in leukemias and lymphomas delineated by fluorescence in situ hybridization Genes Chromosomes Cancer 2000 27 52 58 10564586 10.1002/(sici)1098-2264(200001)27:1<52::aid-gcc7>3.0.co;2-x

[B94] Ko YH Choi KE Han JH Kim JM Ree HJ Comparative genomic hybridization study of nasal-type NK/T-cell lymphoma Cytometry 2001 46 85 91 11309817 10.1002/cyto.1069

[B95] Sun HS Su I-J Lin Y-C Chen J-S Fang S-Y A 2.6 Mb interval on chromosome 6q25.2-q25.3 is commonly deleted in human nasal natural killer/T-cell lymphoma Br J Haematol 2003 122 590 599 12899714 10.1046/j.1365-2141.2003.04419.x

[B96] Nakashima Y Tagawa H Suzuki R Karnan S Karube K Ohshima K Muta K Nawata H Morishima Y Nakamura S Seto M Genome-wide array-based comparative genomic hybridization of natural killer cell lymphoma/leukemia: different genomic alteration patterns of aggressive NK-cell leukemia and extranodal Nk/T-cell lymphoma, nasal type Genes Chromosomes Cancer 2005 44 247 255 16049916 10.1002/gcc.20245

